# Bursts and Isolated Spikes Code for Opposite Movement Directions in Midbrain Electrosensory Neurons

**DOI:** 10.1371/journal.pone.0040339

**Published:** 2012-06-29

**Authors:** Navid Khosravi-Hashemi, Maurice J. Chacron

**Affiliations:** 1 Department of Physiology, McGill University, Montreal, Quebec, Canada; 2 Center for Applied Mathematics in Biology and Medicine, McGill University, Montreal, Quebec, Canada; 3 Department of Physics, McGill University, Montreal, Quebec, Canada; Georgia State University, United States of America

## Abstract

Directional selectivity, in which neurons respond strongly to an object moving in a given direction but weakly or not at all to the same object moving in the opposite direction, is a crucial computation that is thought to provide a neural correlate of motion perception. However, directional selectivity has been traditionally quantified by using the full spike train, which does not take into account particular action potential patterns. We investigated how different action potential patterns, namely bursts (i.e. packets of action potentials followed by quiescence) and isolated spikes, contribute to movement direction coding in a mathematical model of midbrain electrosensory neurons. We found that bursts and isolated spikes could be selectively elicited when the same object moved in opposite directions. In particular, it was possible to find parameter values for which our model neuron did not display directional selectivity when the full spike train was considered but displayed strong directional selectivity when bursts or isolated spikes were instead considered. Further analysis of our model revealed that an intrinsic burst mechanism based on subthreshold T-type calcium channels was not required to observe parameter regimes for which bursts and isolated spikes code for opposite movement directions. However, this burst mechanism enhanced the range of parameter values for which such regimes were observed. Experimental recordings from midbrain neurons confirmed our modeling prediction that bursts and isolated spikes can indeed code for opposite movement directions. Finally, we quantified the performance of a plausible neural circuit and found that it could respond more or less selectively to isolated spikes for a wide range of parameter values when compared with an interspike interval threshold. Our results thus show for the first time that different action potential patterns can differentially encode movement and that traditional measures of directional selectivity need to be revised in such cases.

## Introduction

Motion perception is often required to control animal behavior such as tracking [1]–[5], postural balance [6]–[9] and prey capture [10], [11]. Directional selectivity, in which neurons respond strongly to an object moving in a given direction (‘preferred’) but respond weakly or not at all when the same object moves in the opposite direction (‘null’), is thought to provide a neural correlate of motion perception [12]. Directionally selective neurons have been found in several species including cats [12], rabbits [13], flies [14], and weakly electric fish [15]–[18].

Since the discovery of direction selective neurons [12], several models have been proposed to explain how this selectivity emerges in the brain [19]–[22]. Among these models, so called “Reichardt detectors” have received considerable attention and have been used to describe directional selectivity across several animal species [3], [12]–[14], [18], [23]–[29]. These rely on two fundamental operations to generate directional selectivity [30], [31]: first, asymmetric filtering of information from at least two separate zones within the receptive field generates a directional bias [13], [14], [18], [27], [32], [33] and, second, subsequent nonlinear integration of these inputs [13], [14], [28], [29], [31], [34], [35].

Directional selectivity has been traditionally characterized by comparing the maximum firing rate obtained when a given object moves in a given direction to that obtained when the same object moves in the opposite direction. However, this does not take into account particular action potential patterns. Previous studies have shown that, for stationary stimuli, particular action potential patterns such as bursts (i.e. packets of action potential followed by quiescence) as well as isolated spikes could carry information that is qualitatively different than that carried by the full spike train [36]–[54]. However, whether these action potential patterns carry information about motion direction is poorly understood in general [26], [43].

Weakly electric fish sense distortions of their self-generated electric organ discharge (EOD) via an array of electroreceptor neurons on their skin [55], [56]. These electroreceptors synapse onto pyramidal cells within the hindbrain electrosensory lateral line lobe (ELL), which in turn project to the midbrain torus semicircularis (TS). It was previously shown that TS but not ELL neurons display directionally selective responses to moving objects [18], [35]. The mechanism by which TS neurons generate directionally selective responses has been previously elucidated and is consistent with the Reichardt model. It consists of asymmetric filtering of afferent ELL input across the fish's body surface that is achieved by different time constants of synaptic depression across the receptive field [18] followed by nonlinear integration of these inputs via subthreshold T-type calcium currents [26], [35] (see [55] for review). We have recently found that bursts were more reliable indicators of motion direction than either the full spike or the isolated spike train in TS neurons [26]. These results suggest that isolated spikes actually code for other stimulus features than motion direction. However, a systematic analysis of movement direction coding by bursts and isolated spikes has not been carried out to date.

To address whether isolated spikes can actually code for motion direction, we systematically varied parameters in a previously established model of directional selectivity. Confirming our previous results, we found parameter regimes for which bursts were better indicators of motion direction than either the full spike or the isolated spike trains. However, we also found parameter regimes in which bursts and isolated spikes could both code for movement direction. Specifically, bursts were then preferentially elicited when the object moves in a given direction while isolated spikes were preferentially elicited when the object moves in the opposite direction. Further, our results show that, while the subthreshold T-type calcium conductance was not necessary to observe such regimes, it greatly enhanced the set of parameter values for which they were observed. Experimental recordings from TS neurons confirmed our model's prediction that bursts and isolated spikes can actually code for opposite movement directions. Finally, we considered a plausible neural circuit that can extract isolated spikes from a spike train and quantified this circuit's ability to extract the isolated spikes from a spike train consisting of a mixture of bursts and isolated spikes. Our results show for the first time that different action potential patterns in a given neuron can carry information about different movement directions and suggest that differential coding of stimulus attributes by bursts and isolated spikes is a general feature of sensory processing that is applicable to a wide range of stimuli including motion.

## Results

### Bursts and isolated spikes can code for opposite movement directions

**Figure 1:**
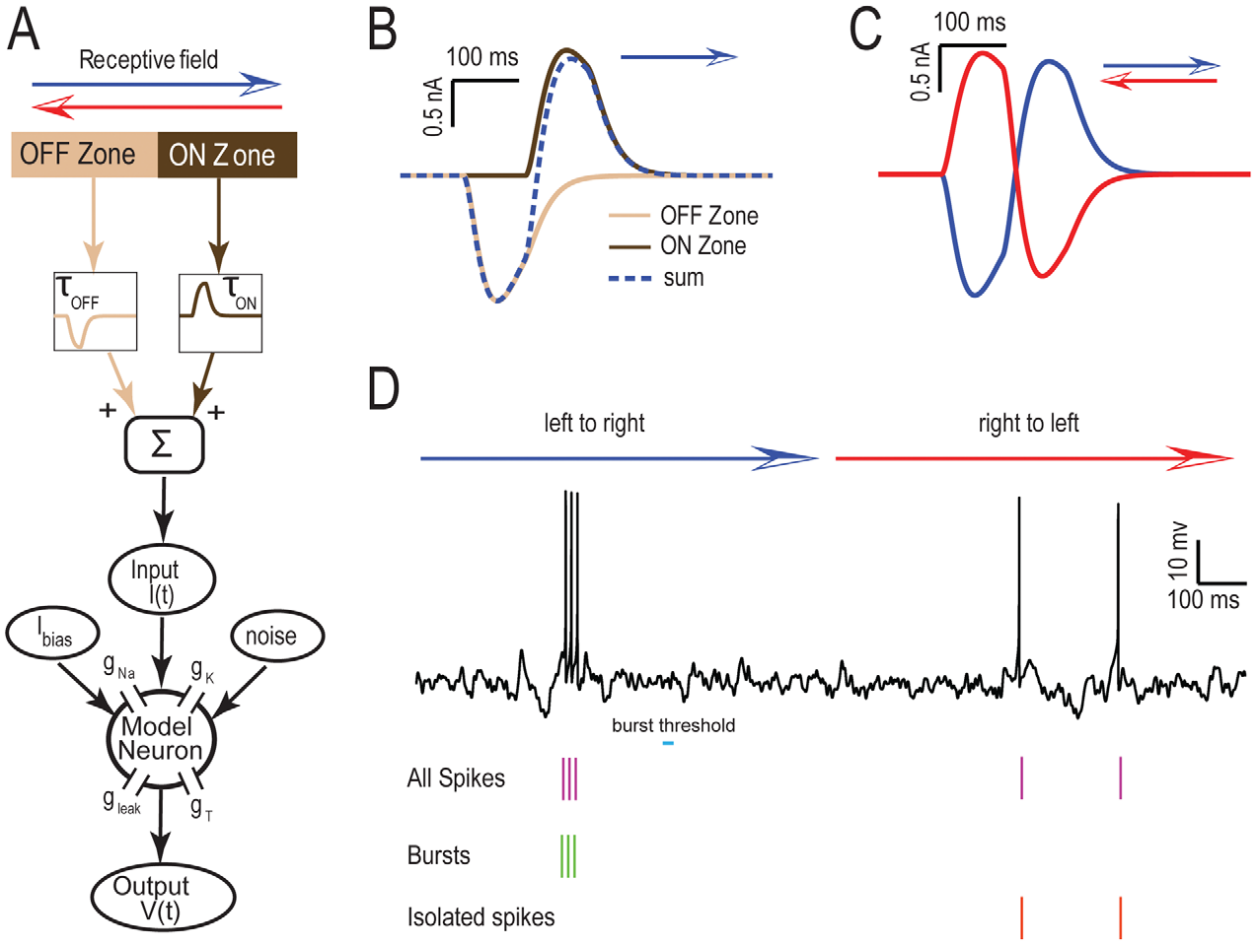
Modeling directional selectivity in TS neurons. **A**) Schematic of our model. The receptive field is composed of two zones: the OFF zone which represents the output of I-type ELL pyramidal cells with synaptic depression time constant τ_OFF_ while the ON zone represents the output of E-type ELL pyramidal cells with synaptic depression time constant τ_ON_. The responses from each zone are then fed into a Hodgkin-Huxley model with spiking sodium (g_Na_), delayed rectifier potassium (g_K_), leak (g_leak_), and T-type calcium (g_T_) conductances. Noise is also added to this model in order to mimic synaptic input from other neurons. **B**) Inputs from OFF zone (beige), ON zone (brown), and the sum of the two (dashed blue) for τ_1_ =  τ_2_ = 500 msec when the object moves from left to right (i.e. from the OFF zone to the ON zone). **C**) Summed input from both zones when the object moves from left to right (blue) and from right to left (red). **D**) Segregating the spike train to bursts and isolated spikes. The top trace is an example membrane potential trace for one trial (object moves from left to right and then from right to left) from our model in response to the inputs shown in C. Spikes (purple lines) belonging to interspike intervals that were less than the burst threshold (cyan) were identified as belonging to bursts (green lines) while those that do not were identified as isolated spikes (red lines).

Our biophysical model is based on the Hodgkin-Huxley formalism [57] (see Materials and Methods). The receptive field is modeled in one dimension as two adjacent zones (ON and OFF) that have time constants of depression τ_ON_ and τ_OFF_, respectively (Fig. 1A). In this model, the OFF zone represents the output of I-type (i.e. inhibited by increases in the stimulus) ELL pyramidal cells and the ON zone represents the output of E-type (i.e. excited by increases in the stimulus) ELL pyramidal cells as both cell types made excitatory connections onto TS neurons [58]. The summed input from each zone is convolved with an alpha function to mimic the synaptic PSP shape and fed into a Hodgkin-Huxley model with leak, spiking sodium, delayed rectifier potassium, and T-type calcium conductances (Fig. 1A, see Materials and Methods). T-type calcium channels are inactivated at resting membrane potential values (i.e. ∼ −60 mV) and require ∼100 ms hyperpolarisation to ∼ −70 mV in order to remove their inactivation after which a subsequent depolarisation will lead to a subthreshold calcium spike, leading to nonlinear integration of synaptic input. Moreover, bursts of sodium action potentials can occur on top of these calcium spikes [59], [60]. However, a simple depolarization from the resting potential will not lead to burst firing as the calcium channel is still inactivated and will instead lead to isolated spike firing [60]. We mimicked the effect of the massive synaptic bombardment that neurons receive under *in vivo* conditions [61], by including a noise term that causes membrane potential fluctuations. This noise term can give rise to a mixture of burst and isolated action potential firing as observed for TS neurons under *in vivo* conditions [26].

The stimulus consists of an object that moves across the receptive field in both directions (see Materials and Methods). Fig. 1B shows the outputs from the ON and OFF zones to this stimulus. When the object moves from left to right (i.e. from the OFF zone to the ON zone), the hyperpolarisation from the OFF zone precedes the depolarization from the ON zone. However, when the object moves in the opposite direction (i.e. from the ON zone to the OFF zone), the depolarisation from the ON zone is truncated by the hyperpolarisation from the OFF zone (Fig. 1C). The membrane potential responses of the model neuron to these moving stimuli are shown in Fig. 1D. When the object moves from left to right, the hyperpolarisation from the OFF zone removes the inactivation of the calcium conductance and the depolarisation from the ON zone activates this conductance, which tends to result in a burst of action potentials (Fig. 1D, top). In contrast, when the object moves in the opposite direction, the depolarisation from ON zone is not preceded by a hyperpolarisation, and thus tends to elicit isolated action potentials (Fig. 1D, top).

We used an ISI threshold criterion to separate the model's output spiketrain into bursts and isolated spikes (Fig. 1D, bottom, see Materials and Methods). Specifically, when a given interspike interval was less than the threshold, the two spikes associated with this interspike interval were considered to belong to a burst [41], [42], [44]. The spikes that were not deemed part of a burst were labelled isolated spikes (Fig. 1D, bottom). We used this criterion to separate the spike train into the burst train (i.e. the train of action potentials that belong to bursts) and the isolated spike train (i.e. the train of action potentials that do not belong to bursts) (see Materials and Methods).

**Figure 2:**
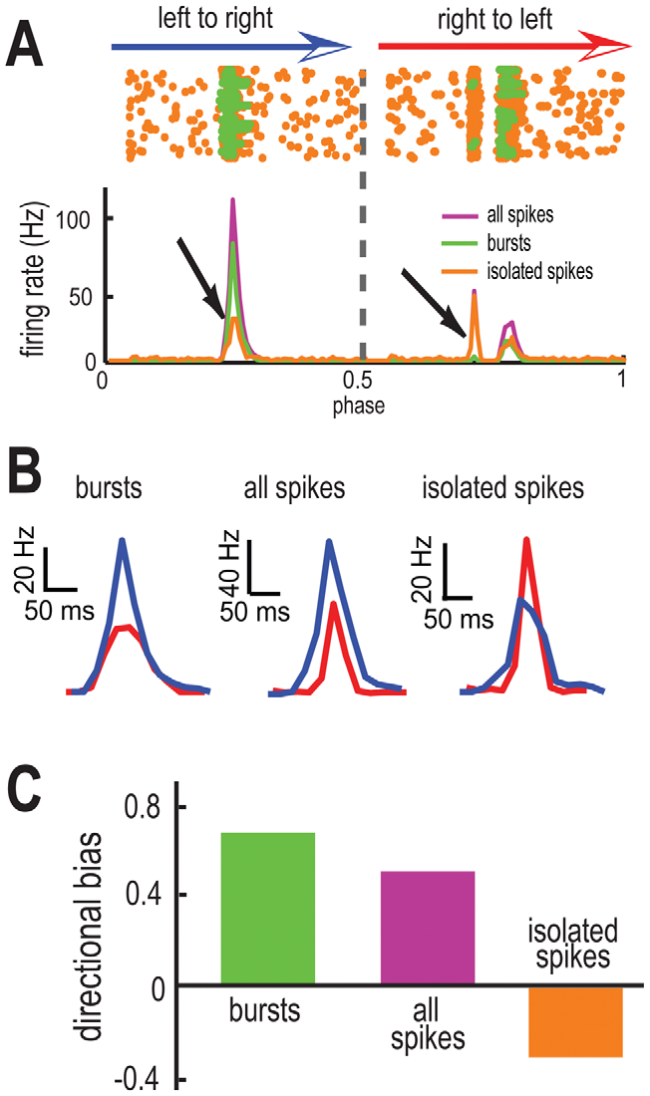
Bursts and isolated spikes code for opposite movement directions. **A**) Raster plot (top) obtained for τ_ON_ = 5 msec, τ_OFF_ = 500 msec from our model. Each dot represents the time at which an action potential occurs. These are color coded as orange for isolated spikes and green for burst spikes. PSTH (bottom) obtained from all spikes (purple), burst spikes (green), and isolated spikes (orange). **B**) PSTH values near the maximum values in the left to right (blue) and right to left (red) directions for burst spikes (left), all spikes (middle), and isolated spikes (right). Note the opposite directional preference of isolated spikes (brown arrow). **C**) Directional biases computed from burst spikes (green), all spikes (purple), and isolated spikes (orange).

The response of our model to this stimulus is presented in Fig. 2. When we used the full spike train to compute the peri-stimulus time histogram (PSTH), the model displayed a strong response when the object moved in the left to right direction and a weaker response when the object moved in the right to left direction (Figs. 2A and 2B, middle). We quantified this difference using a directional bias (DB) index that ranges between −1 and 1 with 0 implying no directional selectivity (see Materials and Methods). Specifically, DB values of 1 and −1 indicate complete direction preference for movement from left to right and from right to left, respectively, while a value of 0 indicates no direction selectivity. We found that this neuron displayed selectivity to the object moving from left to right when using the full spike train (DB = 0.51) (Fig. 2C purple column).

However, qualitatively different results were obtained when we instead used the burst and isolated spike trains to compute the PSTH from this same neuron. We found that bursts mostly occurred when the object moved from left to right (Fig. 2A arrows; Fig. 2C green column), thereby giving rise to a larger directional bias (DB = 0.72) than that of the full spike train. In contrast, isolated spikes mostly occurred when the object moved from right to left (Fig. 2A arrows; Fig. 2C orange column), giving rise to a negative directional bias (DB = −0.34). These results show that bursts and isolated spikes can encode opposite directions of movement.

### Effects of T-type calcium channels on movement direction coding by bursts and isolated spikes

**Figure 3:**
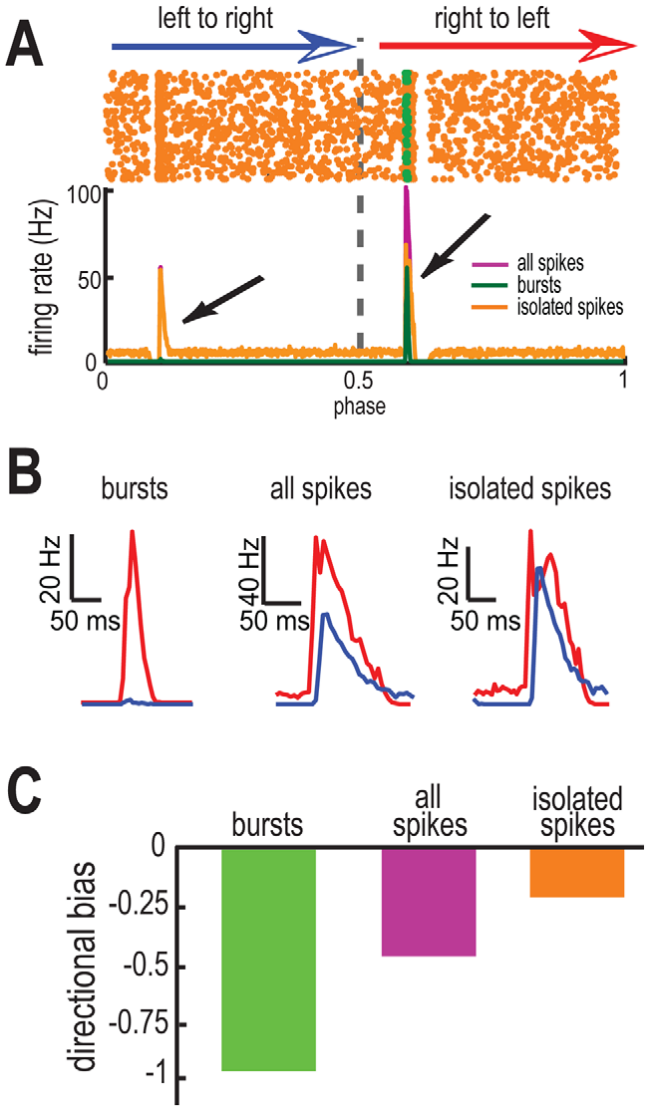
T-type calcium channels promote coding of opposite movement directions by bursts and isolated spikes. **A**) Raster plot (top) obtained for τ_ON_ = 5 msec, τ_OFF_ = 500 msec when g_T_ = 0. The spikes in the raster plot are color coded, as orange for isolated spikes and green for burst spikes. PSTH curves (bottom) obtained from all spikes (purple), burst spikes (green), and isolated spikes (orange). **B**) PSTH values near the maximum values in the left to right (blue) and right to left (red) directions for burst spikes (left), all spikes (center), and isolated spikes (right). **C**) Directional biases computed from all spikes (green), burst spikes (purple), and isolated spikes (orange). We note that the error bars are too small to be shown.

We next investigated movement direction coding by bursts and isolated spikes in our model without the calcium conductance. To do this, we performed numerical simulations of our model with g_T_ = 0. We note that our model then does not generate calcium-mediated burst firing, but can generate short interspike intervals that would be considered as “bursts” according to the ISI threshold criterion when the bias current is sufficiently high. We found that our model displayed a stronger response when the object moved from right to left and a weaker response when the object moved from left to right when the full spike train was used (Fig. 3A, Fig. 3B middle). Our model thus still displayed directional selectivity (DB = −0.46). When we used the burst train, we observed a stronger directional bias (DB = −0.97) as bursts were almost exclusively elicited when the object moves from right to left. In contrast, the isolated spikes tended to be elicited when the object moves in both directions with a slight bias when the object moves from right to left as reflected by a weaker directional bias (DB = −0.21). As such, our results show that both bursts and isolated spikes encoded the same movement direction (i.e. right to left) when we set g_T_ = 0 in our model as they displayed negative directional biases (Fig. 3C).

**Figure 4:**
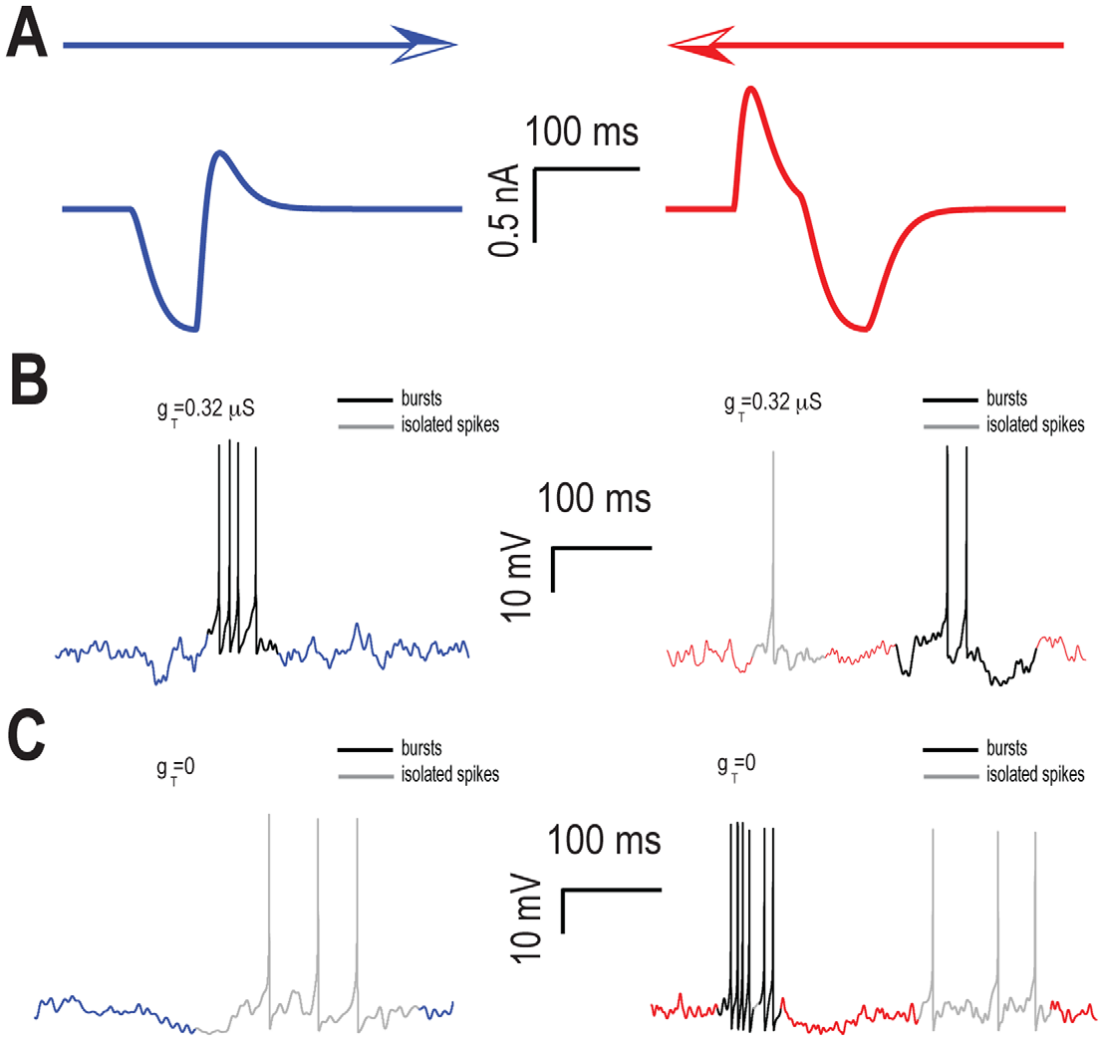
T-type calcium channels promote burst and isolated spike firing when the object moves in opposite directions. **A**) Summed input currents from both zones when the object moves from left to right (blue) and from right to left (red) for τ_ON_ = 5 msec, τ_OFF_ = 500 msec. **B**) Example membrane potential traces when the object moves from left to right (left) and from right to left (right) with the T-type calcium conductance. The response consisted of bursts (black) when the object moved from left to right and of bursts (black) and isolated spikes (gray) when the object moved from right to left. **C**) Example membrane potential traces when the object moves from left to right (blue) and from right to left (red) without the calcium conductance. The response consisted of isolated spikes (gray) when the object moved from left to right and of bursts (black) and isolated spikes (gray) when the object moved from right to left.

In order to better understand these results, we then plotted the inputs to the model when the object moves from left to right (Fig. 4A, left) and right to left (Fig. 4A. right). In the left to right direction, the hyperpolarisation from the OFF zone attenuates the subsequent depolarisation from the ON zone (Fig. 4A, left). In contrast, in the right to left direction, the initial depolarisation from the ON zone is truncated by the subsequent hyperpolarisation from the OFF zone (Fig. 4A, right). The response of our model to these different inputs strongly depends on the value of the T-type conductance g_T_. When g_T_ is present, the initial hyperpolarisation from the OFF zone removes the inactivation of this conductance and the subsequent depolarization activates it, thereby causing a burst of action potentials as explained above when the object moves from left to right (Fig. 4B, left). In contrast, the initial depolarisation gives rise to isolated spikes when the object moves from right to left as the T-type conductance is then inactivated (Fig. 4B, right). The following hyperpolarisation only partially removes this inactivation and the subsequent repolarization gives rise to a burst of action potentials albeit with a larger intraburst interval (Fig. 4B, right). Therefore, our model tends to respond with a mixture of bursts and isolated spikes when the object moves from right to left.

Qualitatively different results were seen when we removed the T-type conductance (i.e. g_T_ = 0). When the object moves from left to right, the depolarization from the ON zone is partially occluded by the preceding hyperpolarisation from the OFF zone and thus gives rise to isolated spiking (Fig. 4C, left). When the object moves from right to left, the initial depolarisation from the ON zone gives rise to a burst of action potentials. The subsequent hyperpolarization from the OFF zone silences spiking and the repolarisation then gives rise to isolated spikes (Fig. 4C, right). As such, our model gives rise to isolated spikes when the object moves in both directions and to bursts preferentially when the object moves from right to left.

### Exploring the effect of the synaptic depression time constants on movement direction coding by bursts and isolated spikes

**Figure 5:**
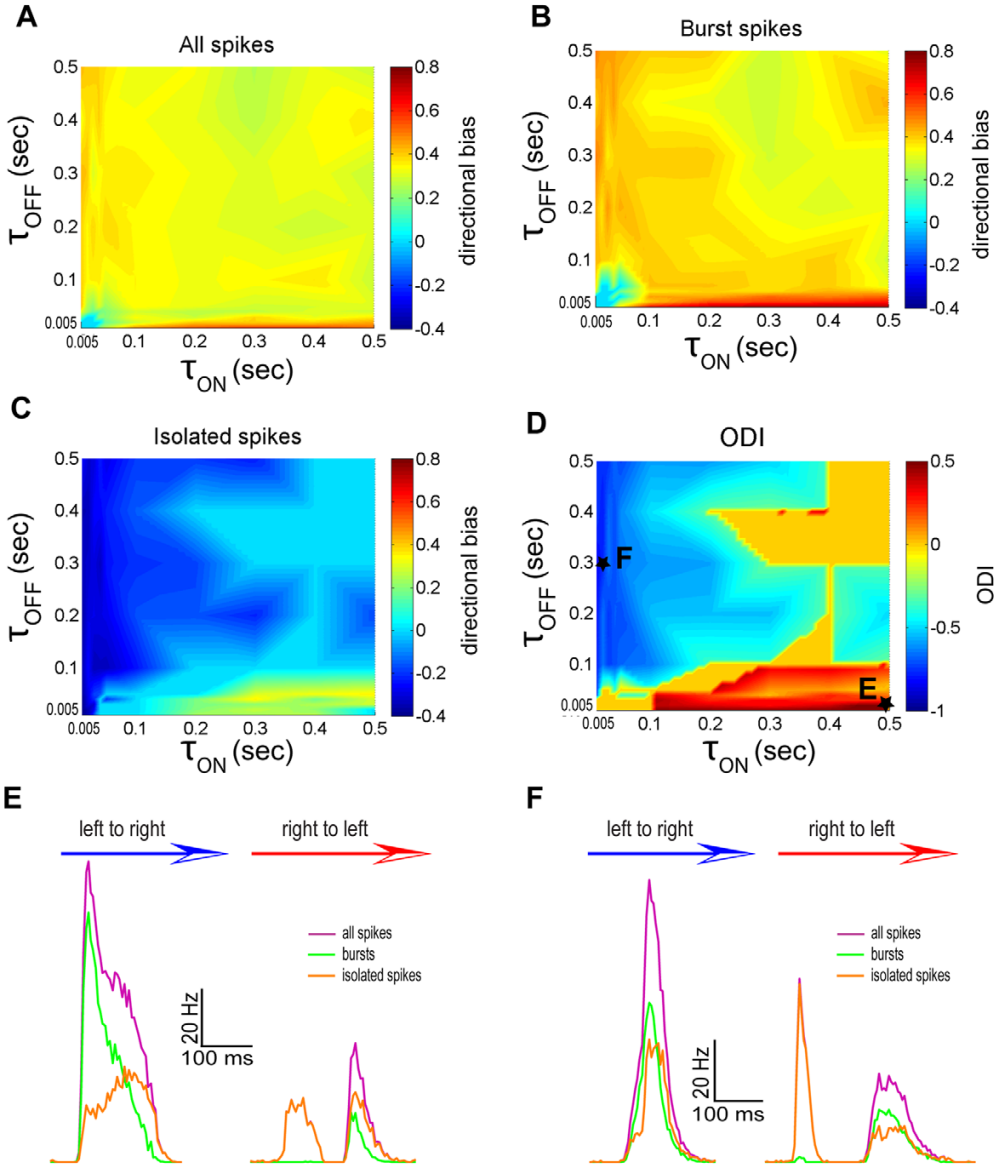
The synaptic depression time constants τ_ON_ and τ_OFF_ strongly influence movement direction coding by bursts and isolated spikes. **A**) Directional bias computed from the full spike train as a function of τ_ON_ and τ_OFF_. **B**) Directional bias computed from the burst spike train as a function of τ_ON_ and τ_OFF_. **C**) Directional bias computed from the isolated spike train as a function of τ_ON_ and τ_OFF_. **D**) Opposite direction selectivity index (ODI) as a function of τ_ON_ and τ_OFF_. **E**) PSTH values near the maximum values in the left to right (blue arrow) and right to left (red arrow) directions for the full spike (purple), burst (green), and isolated (orange) spike trains for an example data point marked with a star in panel D. **F**) PSTH values near the maximum values in the left to right (blue arrow) and right to left (red arrow) directions for the full spike (purple), burst (green), and isolated (orange) spike trains for another example data point marked with a star in panel D.

We then systematically varied model parameters and characterized the directional biases of bursts and isolated spikes with the T-type conductance present. We first varied the synaptic depression time constants from the ON (τ_ON_) and OFF (τ_OFF_) zones in our model. Our results show that varying these can lead to dramatic qualitative differences between the directional biases of bursts and isolated spikes. Indeed, for small τ_OFF_ and large τ_ON_ values (i.e. τ_OFF_ <0.1 sec and τ_ON_ >0.1 sec), the full (Fig. 5A), burst (Fig. 5B), and isolated (Fig. 5C) spike trains all displayed positive directional biases and thus encoded the same movement direction. However, the directional bias of isolated spikes was smaller in magnitude than that of the full and burst trains, which corresponds to the regime described in our previous study [26]. We will henceforth refer to this regime as “same direction selectivity”. In contrast, for large τ_OFF_ and small τ_ON_ values (i.e. τ_OFF_ >0.1 sec and τ_ON_ <0.1 sec), both the full (Fig. 5A) and burst (Fig. 5B) trains displayed a positive directional bias while the isolated spike train (Fig. 5C) displayed a negative directional bias. We will henceforth refer to this regime as “opposite direction selectivity”.

In order to better characterize both regimes, we computed an opposite directionality index (ODI, see Materials and Methods). This index is positive when the directional biases of both bursts and isolated spikes have the same sign, negative when they are opposite in sign, and 0 when one does not display significant directional selectivity. We found that the ODI was positive for small τ_OFF_ and large τ_ON_ values (i.e. τ_OFF_ <0.1 sec and τ_ON_ >0.1 sec) and negative for large τ_OFF_ and small τ_ON_ values (i.e. τ_OFF_ >0.1 sec and τ_ON_ <0.1 sec) (Fig. 5D).

In order to better understand why varying the depression time constants τ_ON_ and τ_OFF_ can give rise to qualitatively different regimes, we plotted the PSTH curves for the full, burst, and isolated spike trains for two sets of parameter values that gave rise to same and opposite direction selectivity regimes in Figs. 5E and 5F, respectively. The parameter values used for the same and opposite direction selectivity regimes are shown in Fig. 5D as points “E” and “F”, respectively. For the same direction selectivity regime, the maximum firing rate from the full, burst, and isolated spike trains was strongest when the object moves from left to right (Fig. 5E). In contrast, for the opposite directional selectivity regime, the maximum firing rate for the full spike and burst trains were higher when the object moves from left to right while that of isolated spike train is highest when the object moves from right to left (Fig. 5F).

We thus conclude that the ratio τ_OFF_/τ_ON_ has a strong influence on whether bursts and isolated spikes code for the same or opposite movement directions. Indeed, the former regime tended to occur for low values of τ_OFF_/τ_ON_ while the latter regime tended to occur for high values of τ_OFF_/τ_ON_. We also varied the gains from the ON and OFF zones, G_ON_ and G_OFF_, and found that varying these gave rise to qualitatively similar results in that opposite movement direction regimes were mostly seen for high values of G_OFF_/G_ON_ (Fig. S1).

### Exploring the effect of bias current on movement direction coding by bursts and isolated spikes

**Figure 6:**
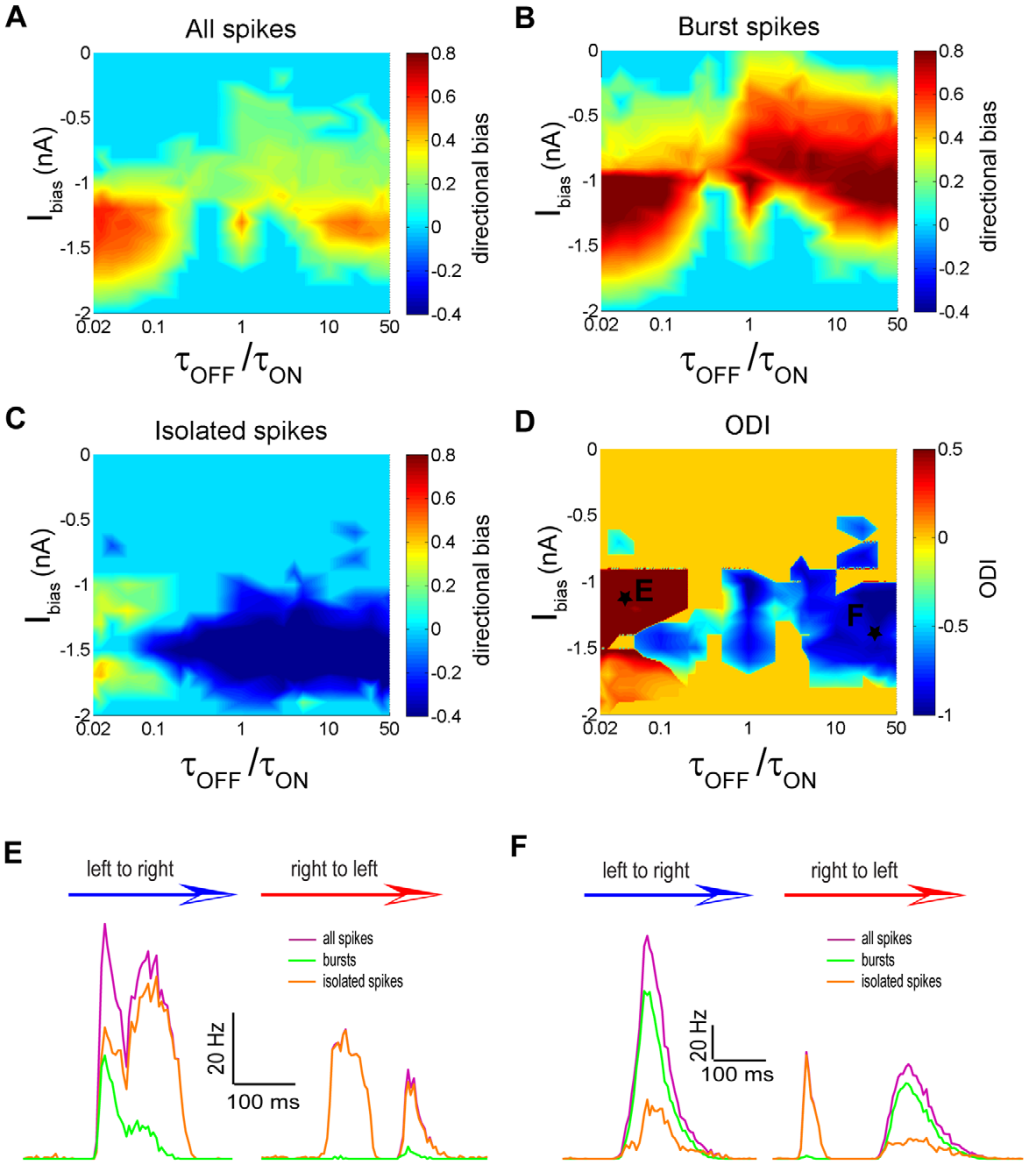
The bias current I_bias_ and synaptic depression time constant ratio τ_OFF_/τ_ON_ strongly influence movement direction coding by bursts and isolated spikes. **A**) Directional bias computed from the full spike train as a function of τ_OFF_/τ_ON_ and I_bias_. **B**) Directional bias computed from the burst train as a function of τ_OFF_/τ_ON_ and I_bias_. **C**) Directional bias computed from the isolated spike train as a function of τ_OFF_/τ_ON_ and I_bias_. **D**) Opposite direction selectivity index as a function of τ_OFF_/τ_ON_ and I_bias_. **E**) PSTH values near the maximum values in the left to right (blue arrow) and right to left (red arrow) directions for the full spike (purple), burst (green), and isolated (orange) spike trains for an example data point marked with a star in panel D. **F**) PSTH values near the maximum values in the left to right (blue arrow) and right to left (red arrow) directions for the full spike (purple), burst (green), and isolated (orange) spike trains for another example data point marked with a star in panel D.

We next explored whether the bias current I_bias_ influenced coding of movement direction by bursts and isolated spikes. To do so, we plotted the directional biases of the full (Fig. 6A), burst (Fig. 6B), and isolated (Fig. 6C) spike trains as a function of both the bias current I_bias_ and the ratio of the synaptic depression time constants τ_OFF_/τ_ON_ which was varied so as to observe both same and opposite direction selectivity regimes (see Materials and Methods). Our results show that when I_bias_ was low (i.e. <−1.8 nA) or high (i.e. >−0.5 nA), neither bursts (Fig. 6B) nor isolated spikes (Fig. 6C) displayed significant directional selectivity, resulting in an ODI of zero (Fig. 6D). Regimes in which the ODI was non-zero tended to occur for intermediate values of I_bias_ (i.e. −1.8 nA<I_bias_<−0.5 nA). For low values of τ_OFF_/τ_ON_ (i.e. τ_OFF_/τ_ON_<0.2), we observed same directional selectivity regimes characterized by positive ODI (Fig. 6D). In contrast, for high values of τ_OFF_/τ_ON_ (i.e. τ_OFF_/τ_ON_>0.2), we observed regimes of opposite direction selectivity characterized by negative ODI (Fig. 6D). In particular, we found that, for some parameter values (i.e. I_bias_ = −1 nA and τ_OFF_/τ_ON_ = 1), the full spike train displayed weak directional selectivity (Fig. 6A) while both the burst (Fig. 6B) and isolated (Fig. 6C) spike trains displayed strong selectivity for opposite movement directions. We return to this point below in the discussion.

The PSTH curves for the full, burst, and isolated spike trains are shown for two sets of parameter values that gave rise to same and opposite direction selectivity regimes in Figs. 6E and 6F, respectively. The parameter values used for the same and opposite direction selectivity regimes are shown in Fig. 6D as points “E” and “F”, respectively. For the same direction selectivity regime, the maximum firing rate from the full spike, burst, and isolated spike trains was strongest when the object moves from left to right (Fig. 6E). On the other hand, for the opposite directional selectivity regime, the maximum firing rates for the full spike and the burst trains were both greatest when the object moves from left to right while that of isolated spikes was greatest when the object moves from right to left (Fig. 6F).

### T-type calcium currents promote coding of opposite movement directions by bursts and isolated spikes

**Figure 7:**
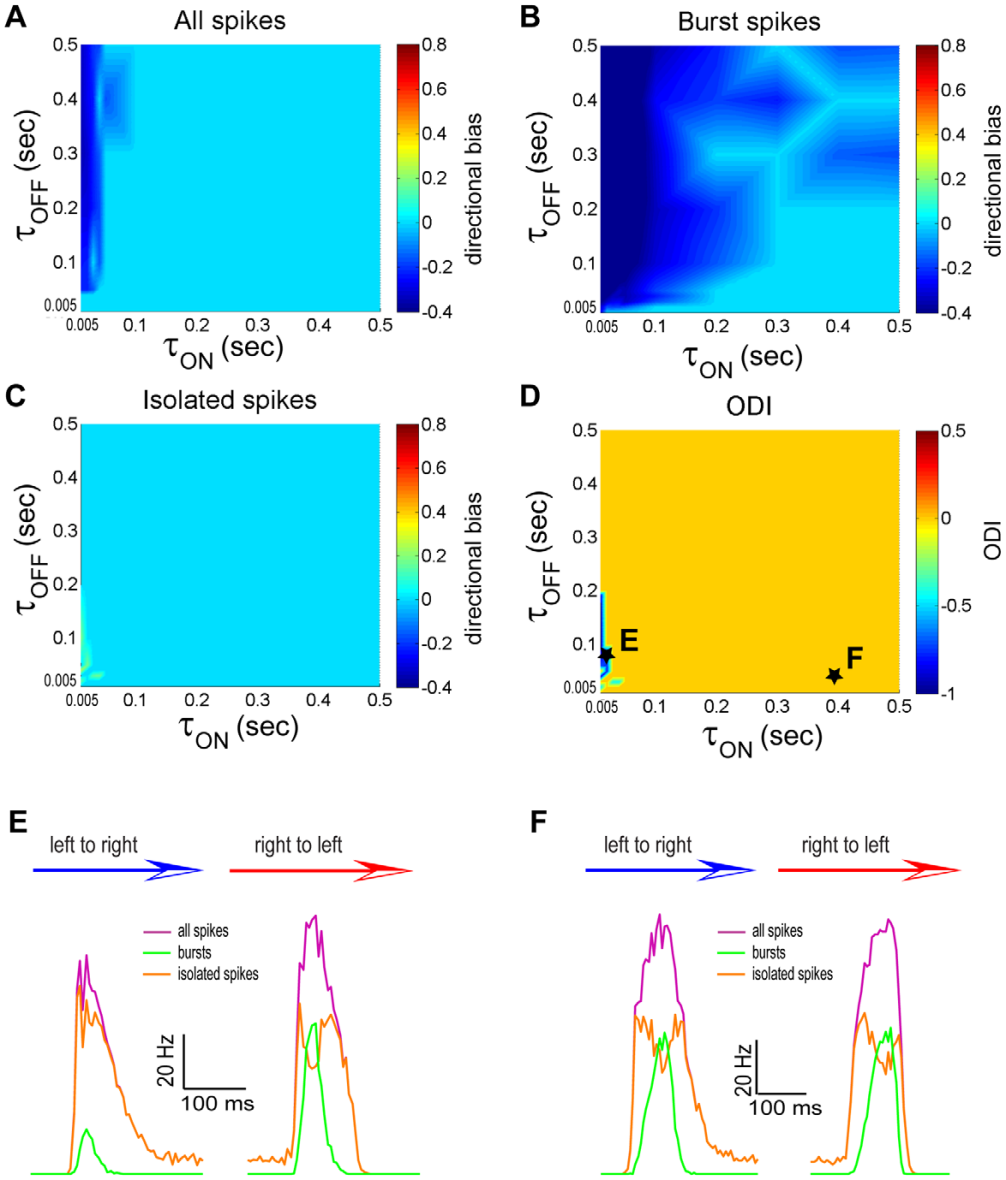
The synaptic depression time constants τ_ON_ and τ_OFF_ influence movement direction coding by bursts and isolated spikes with g_T_ = 0. **A**) Directional bias computed from the full spike train as a function of τ_ON_ and τ_OFF_. **B**) Directional bias computed from the burst spike train as a function of τ_ON_ and τ_OFF_. **C**) Directional bias computed from the isolated spike train as a function of τ_ON_ and τ_OFF_. **D**) Opposite direction selectivity index as a function of τ_ON_ and τ_OFF_. **E**) PSTH values near the maximum values in the left to right (blue arrow) and right to left (red arrow) directions for the full spike (purple), burst (green), and isolated (orange) spike trains for an example data point marked with a star in panel D. **F**) PSTH values near the maximum values in the left to right (blue arrow) and right to left (red arrow) directions for the full spike (purple), burst (green), and isolated (orange) spike trains for another example data point marked with a star in panel D.

We next explored how different parameters influenced movement coding by bursts and isolated spikes in our model without the T-type calcium conductance (i.e. g_T_ = 0). When using the full spike train, we obtained directional bias values that were negative for low values of τ_ON_ (i.e. τ_ON_<0.1 sec) and zero otherwise (Fig. 7A). In contrast, when using the burst train, we obtained directional bias values that were near zero when τ_ON_ was large (i.e. τ_ON_>0.2 sec) and τ_OFF_ was small (i.e. τ_OFF_<0.2 sec) and negative otherwise (Fig. 7B). The isolated spike train (Fig. 7C) tended to display directional bias values near zero except for low values of τ_ON_ (i.e. τ_ON_<0.02 sec) and τ_OFF_ (i.e. τ_OFF_<0.2 sec) where it was positive. As such, the ODI was zero for almost all values of τ_ON_ and τ_OFF_ except for low values of τ_ON_ (i.e. τ_ON_<0.02 sec) and τ_OFF_ (i.e. τ_OFF_<0.2 sec) for which it was negative (Fig. 7D). We also varied the gains G_ON_ and G_OFF_ and found qualitatively similar results in that the parameter regions for which opposite directional selectivity was observed were greatly reduced (compare Figs. S2 and S1).

The PSTH curves for the full spike train, bursts, and isolated spikes are shown for parameter values for which the ODI was negative and null in Figs. 7E and 7F, respectively. These values correspond to those indicated by the points “E” and “F” in Fig. 7D. In the regime where the opposite directional selectivity regime was observed, the firing rates from the full spike and burst trains were both greatest when the object moves from right to left while the maximum firing rate from the isolated spike train was greatest when the object moves from left to right (Fig. 7E). In contrast, in the regime where no directional selectivity was observed, the maximum firing rates of the full, burst, and isolated spike trains were all approximately equal for both movement directions (Fig. 7F).

**Figure 8:**
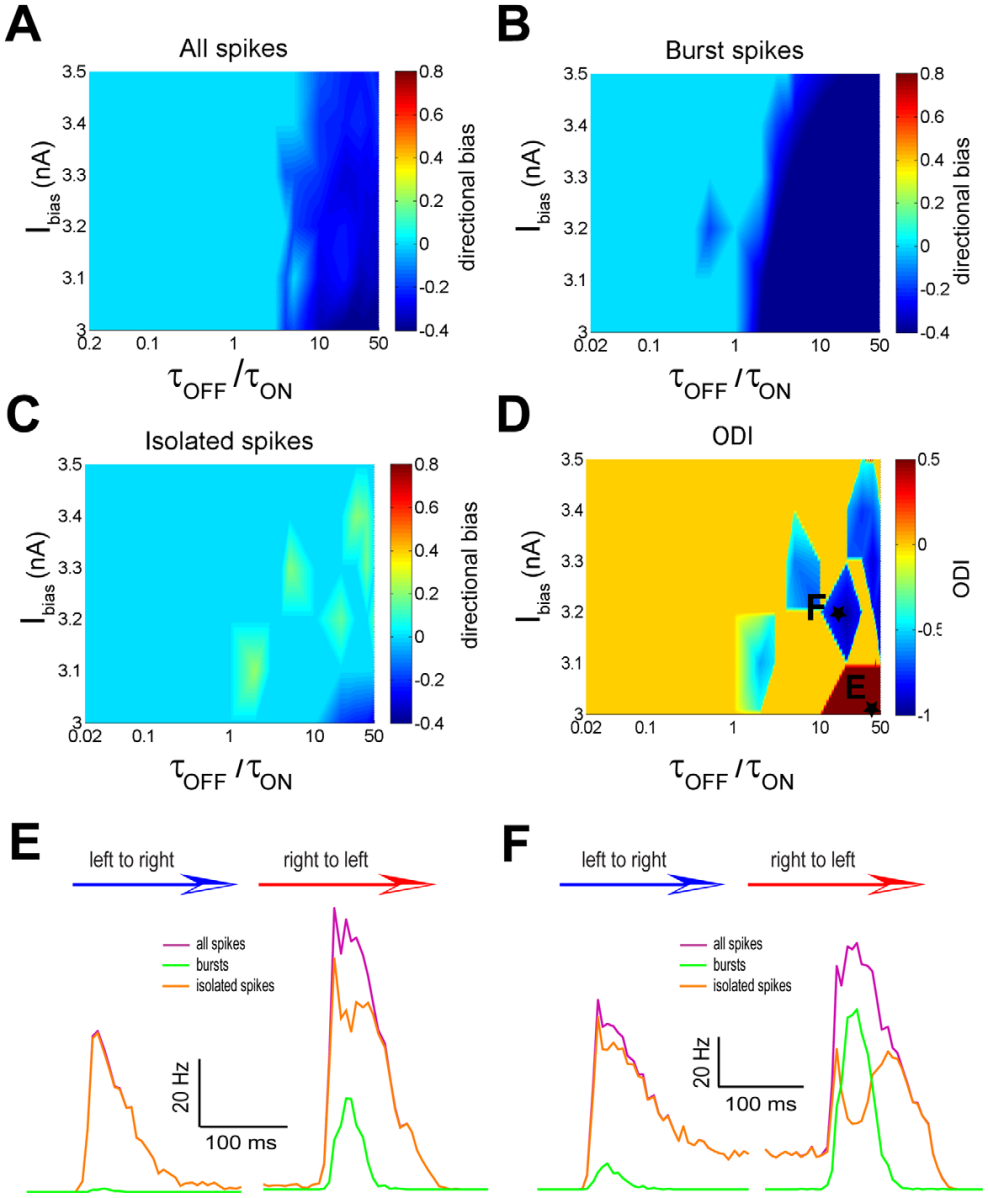
The bias current I_bias_ and synaptic depression time constant ratio τ_OFF_/τ_ON_ influence movement direction coding by bursts and isolated spikes with g_T_ = 0. **A**) Directional bias computed from the full spike train as a function of τ_OFF_/τ_ON_ and I_bias_. **B**) Directional bias computed from the burst train as a function of τ_OFF_/τ_ON_ and I_bias_. **C**) Directional bias computed from the isolated spike train as a function of τ_OFF_/τ_ON_ and I_bias_. **D**) Opposite direction selectivity index as a function of τ_OFF_/τ_ON_ and I_bias_. **E**) PSTH values near the maximum values in the left to right (blue arrow) and right to left (red arrow) directions for the full spike (purple), burst (green), and isolated (orange) spike trains for an example data point marked with a star in panel D. **F**) PSTH values near the maximum values in the left to right (blue arrow) and right to left (red arrow) directions for the full spike (purple), burst (green), and isolated (orange) spike trains for another example data point marked with a star in panel D.

We next plotted the directional biases of the full (Fig. 8A), burst (Fig. 8B), and isolated (Fig. 8C) spike trains as a function of both the bias current I_bias_ and the ratio of the synaptic depression time constants τ_OFF_/τ_ON_ when g_T_ = 0. Our results show that the bias current I_bias_ can significantly influence movement direction coding by the full, burst, and isolated spike trains (Figs. 8A, B, C). Indeed, both the full spike (Fig. 8A) and burst (Fig. 8B) trains displayed similar profiles: no directional selectivity was observed for low values of τ_OFF_/τ_ON_ (i.e. τ_OFF_/τ_ON_<3) and negative directional biases were observed for higher values. In contrast, the isolated spike train (Fig. 8C) displayed a qualitatively different profile in that negative directional biases where observed for high values of τ_OFF_/τ_ON_ (i.e. τ_OFF_/τ_ON_>10) and low bias current values (i.e. I_bias_<3.1 nA) while positive values were observed for larger bias current values (i.e. I_bias_>3.1 nA) (Fig. 8C). As a result, the opposite directional selectivity index ODI displayed both positive and negative values when plotted as a function of I_bias_ and τ_OFF_/τ_ON_ (Fig. 8D). As such, we observed both same and opposite direction selectivity regimes in our model without the T-type conductance.

The PSTH curves for the full spike train, bursts, and isolated spikes are shown for example same and opposite direction selectivity regimes in Figs. 8E and 8F, respectively. The parameter values used for the same and opposite direction selectivity regimes are shown in Fig. 8D as points “E” and “F”, respectively. For the same direction selectivity regime, the maximum firing rate from the full spike, burst, and isolated spike trains is strongest when the object moves from right to left (Fig. 8E). On the other hand, for the opposite directional selectivity regime, the maximum firing rate for the full spike and the burst trains are higher when the object moves from right to left while that of isolated spikes is highest when the object moves from left to right (Fig. 8F).

These results show that bursting mediated by T-type calcium channels is not necessary to observe opposite direction selectivity. However, such bursting greatly extends the range of values of the synaptic time constants τ_ON_ and τ_OFF_ and the bias current I_bias_ for which such coding is observed. We also note that the magnitude of directional biases observed for either of the full, burst, and isolated spike trains was smaller overall without the T-type conductance (compare Figs. 5 and 7 as well as Figs. 6 and 8). We conclude that T-type calcium channels promote movement coding by bursts and isolated spikes.

### Electrosensory midbrain neurons display opposite coding of movement direction by bursts and isolated spikes

**Figure 9:**
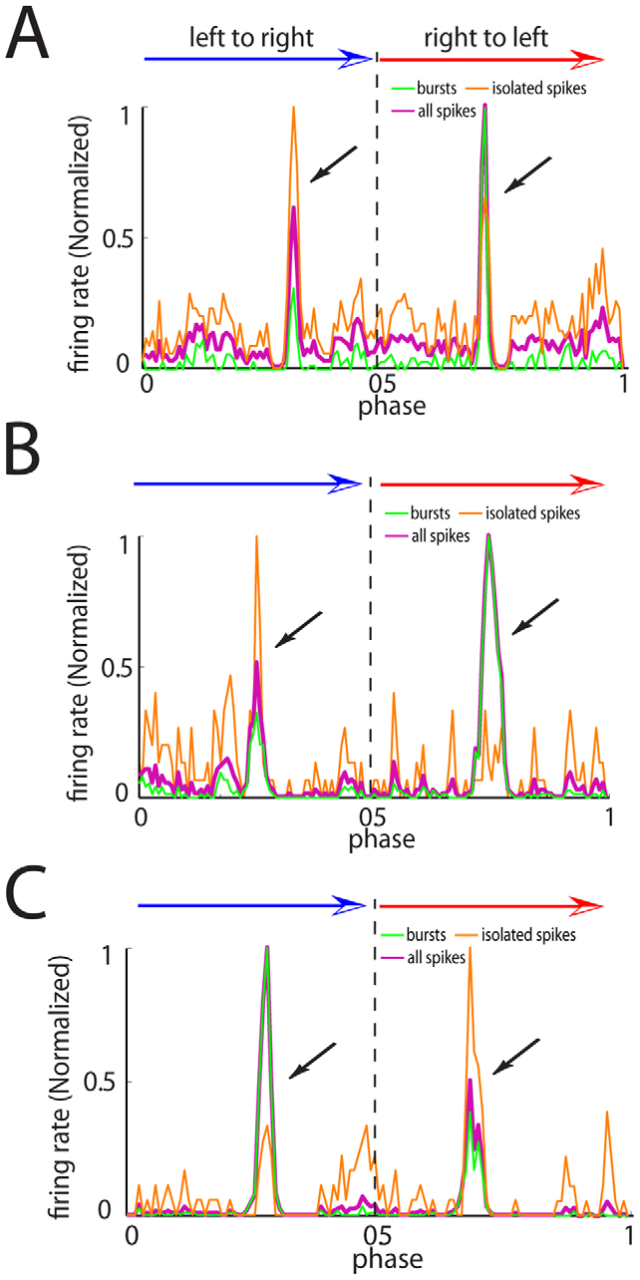
Electrosensory midbrain neurons can display opposite movement direction coding by bursts and isolated spikes. **A**) Peri-stimulus time histogram (PSTH) for an example neuron computed from all spikes (purple), bursts (green), and isolated spikes (orange). The curves have been normalized by their maximum values. Directional bias (DB) values were −0.64, −0.39, and 0.36 for burst, all spikes, and isolated spikes, respectively. **B**) PSTH for another example neuron computed from all spikes (purple), bursts (green), and isolated spikes (orange). The curves have been normalized to 1. Directional bias (DB) values were −0.59, −0.5, and 0.56 for burst, all spikes, and isolated spikes, respectively. **C**) PSTH for another example neuron computed from all spikes (purple), bursts (green), and isolated spikes (orange). The curves have been normalized to 1. Directional bias (DB) values were 0.6, 0.5, and −0.63 for burst, all spikes, and isolated spikes, respectively.

Our analysis of the effects of different parameters on movement direction coding by bursts and isolated spikes has shown the existence of regimes for which bursts and isolated spikes code for the same movement direction and regimes for which bursts and isolated spikes code for opposite movement directions. In order to test this prediction, we performed extracellular recordings from N = 32 TS neurons *in vivo* while moving an object back and forth along the rostro-caudal axis of the animal as done previously [18], [26], [35], [62] (see Materials and Methods). We found that bursts and isolated spikes could code for opposite movement directions in 3 neurons. The PSTH obtained for the full, burst, and isolated spike trains for these three neurons are shown in Figs. 9A, 9B, 9C. We found that opposite coding of movement direction by bursts and isolated spikes was most pronounced for the neuron from Fig. 9C. Indeed, this neuron responded mostly with bursts when the object moved from tail to head and responded mostly with isolated spikes when the same object moved from head to tail (Fig. 9C). This was reflected in the directional biases from the burst and isolated spike trains that were 0.6, and −0.63, respectively. As such, bursts and isolated spikes displayed directional biases that were almost equal in magnitude for this neuron. These data suggest that there exists neurons in TS for which bursts and isolated spikes can code for opposite movement directions.

### Decoding isolated spikes using a delay mechanism coupled with inhibition

Any information carried by action potential patterns such as bursts and isolated spikes is only functionally relevant if it is decoded by downstream neurons. We have previously proposed a biologically plausible circuitry for extracting burst spikes [26]. However, plausible neural circuits that can selectively respond to isolated spikes but are insensitive to bursts have not been proposed to date. We note that the ISI threshold criterion that we have used to separate bursts and isolated spikes is acausal in nature This is because any given spike can only be classified as being part of a burst based on whether the next spike occurs after an interval of time that is less than the burst threshold. Similarly, any given spike can only be classified as isolated if the next spike occurs after an interval of time that is greater than the burst threshold.

**Figure 10:**
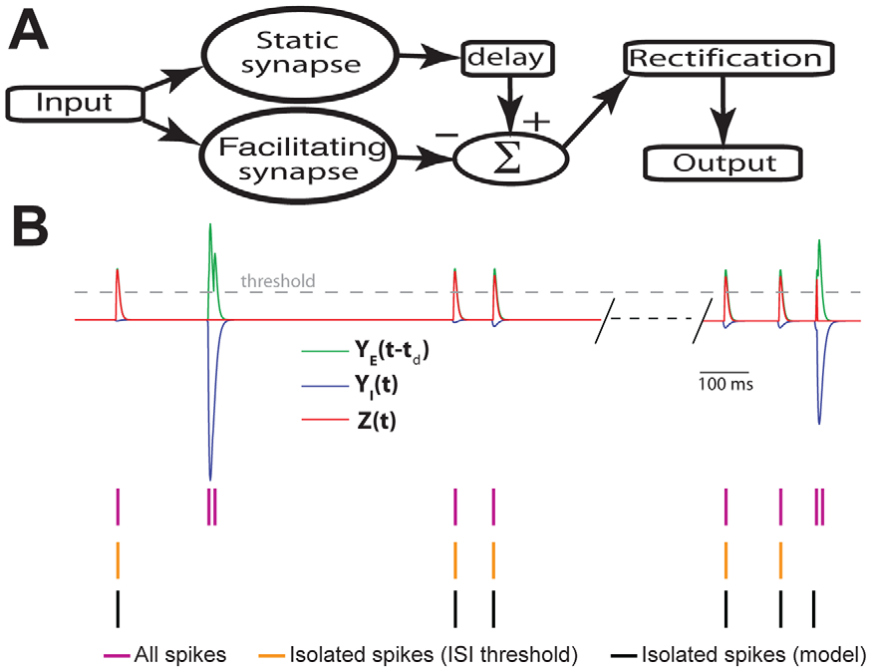
A biophysically plausible neural circuit can accurately extract isolated spikes and therefore decode their information about movement direction. **A**) Schematic of the decoding model for isolated spikes. It consists of parallel processing by two synapses with one displaying facilitation and the other displaying no plasticity (i.e. “static”). The output from the static synapse Y_E_(t) is delayed and the output from the facilitating synapse Y_I_(t) is then subtracted from it. This signal is then half-wave rectified to give the output Z(t). Finally, Z(t) is thresholded to obtain the output spikes. **B**) Performance of the decoding model compared with the performance of an ISI threshold criterion at detecting isolated spikes. Shown are the delayed output of the static excitatory synapse Y_E_(t−t_d_) (green trace), facilitating inhibitory synapse Y_I_(t) (blue trace), and the output of the model Z(t) (red trace) with the threshold used to detect output spikes (dashed gray trace), the original spike train (purple ticks), the isolated spikes according to the ISI threshold (orange ticks), and the isolated spikes according to the decoding model (black ticks). Parameter values used were τ_F_ = 200 msec, τ_D_ = 500 msec, τ_E_ = 5 msec, τ_I_ = 8 msec, G_I_ =  5, I_0_ = 3.41 msec, t_d_ = 4 msec.

A schematic of a biophysically plausible neural circuit that is sensitive to isolated spikes is shown in Figure 10A (see Materials and Methods). It consists of two synapses: the first is excitatory and displays no synaptic plasticity (i.e. the EPSP amplitude elicited by each presynaptic action potential is the same), and the second is inhibitory and displays strong short-term facilitation. The second synapse, therefore, responds preferentially to bursts as shown previously [26]. The output of the excitatory synapse is delayed with respect to the output of the inhibitory synapse, and both inputs are then summed and half-wave rectified (Fig. 10A). Intuitively, this circuit should be sensitive to isolated spikes for the following reason: bursts will give rise to greater facilitation of the inhibitory synapse, thereby causing a larger inhibition in the postsynaptic cell that will tend to prevent a response to the bursts from the excitatory synapse due to the delay. In contrast, isolated spikes will not induce such facilitation. As a result the inhibition is sufficiently low such that the excitation from the first synapse can reach threshold for spiking. We note that such a scheme is not unreasonable since inhibition can sometimes precede excitation in midbrain neural circuits [63], [64].

**Figure 11:**
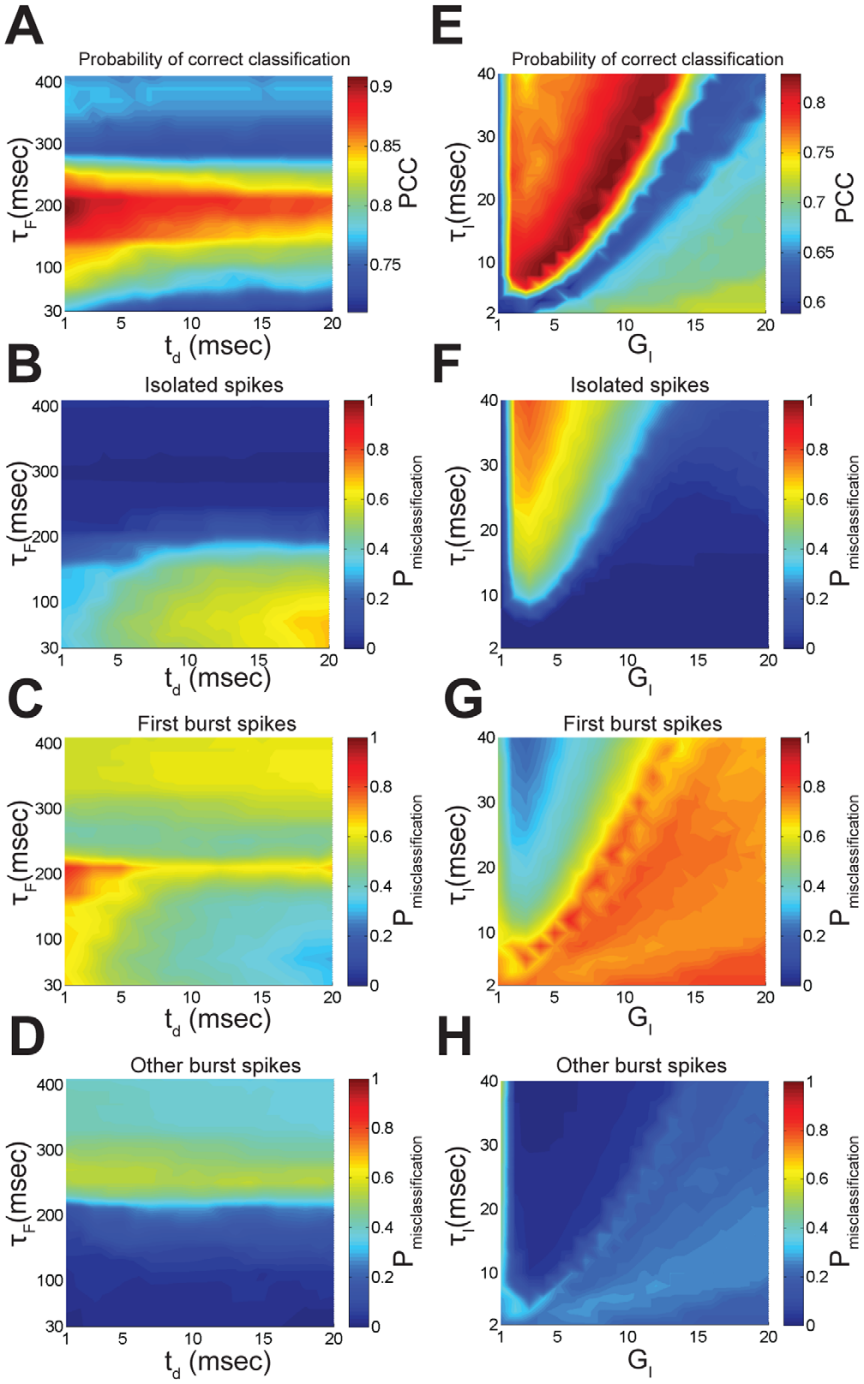
Extracting isolated spikes using a biologically plausible model. **A**) Probability of correct classification PCC as a function of the facilitation time constant τ_f_ and delay t_d_. **B, C, D** Probability of misclassification P_misclassification_ for the spikes that, according to the ISI threshold, were considered to be isolated spikes (**B**), the first spikes of a burst (**C**), or any other spikes of a burst (**D**), as a function of the facilitation time constant τ_f_ and delay t_d_. Other parameter values used were τ_D_ = 500 msec, τ_E_ = 5 msec, τ_I_ = 5 msec, G_I_ = 7, I_0_ = 3.41 msec. **E**) Probability of correct classification PCC as a function of the inhibition time constant τ_I_ and gain G_I_. **F, G, H** Probability of misclassification P_misclassification_ for the spikes that, according to the ISI threshold, were considered to be isolated spikes (**F**), the first spikes of a burst (**G**), or any other spikes of a burst (**H**), as a function of the inhibition time constant τ_I_ and gain G_I_. Other parameter values used were τ_F_ = 200 msec, τ_D_ = 500 msec, τ_E_ = 5 msec, I_0_ = 3.41 msec, t_d_ = 4 msec.

We next tested the performance of this simple model in segregating isolated spikes from bursts to that of an ISI threshold. Our results show that this model was accurate at detecting isolated spikes (Fig. 10B). The spikes that were incorrectly classified tended to be the first spikes of bursts as determined by the ISI threshold that occurred after a period of isolated spiking, as the inhibition is then too weak and too short to block these (Fig. 10B). We then quantified this performance by using signal detection theory [65] (see Materials and Methods) and found that this model gave high probabilities of correct classification for a wide range of delay t_d_ and synaptic facilitation time constant τ_F_ values (Fig. 11A). We also investigated whether the incorrectly classified spikes actually belonged to bursts and isolated spikes as determined by the ISI threshold. To do so, we plotted the probability of misclassification for spikes that, according to the ISI threshold, were considered isolated spikes (Fig. 11B), the first spike of a burst (Fig. 11C), or any other spike of a burst (Fig. 11D). Thus, our results show that, for the parameter values that gave rise to the maximum probability of correct classification, the majority (≈90%) of spikes that were incorrectly classified were actually the first spikes of a burst for the reason mentioned above. We found that these percentages strongly depended on parameter values (Figs. 11B, C, D). For example, increasing the delay for a given value of the facilitation time constant reduces the percentage of misclassified first spikes of a burst (Fig. 11C), increases the percentage of misclassified isolated spikes (Fig. 11B), and does not affect the remaining percentage of misclassified spikes that are part of a burst (Fig. 11D), but decreases the probability of correct classification (Fig. 11A).

We next varied both the inhibition time constant τ_I_ and gain G_I_ in our model. Our results show that the maximum probability of correct classification could be obtained for a wide range of values (Fig. 11E). Again, for the parameter values that gave rise to maximum probability of correct classification, the majority of misclassified spikes were actually the first spikes of bursts as seen by plotting the percentage of misclassified spikes that were considered isolated spikes (Fig. 11F), the first spike of a burst (Fig. 11G), or any other spike of a burst (Fig. 11H).

**Figure 12:**
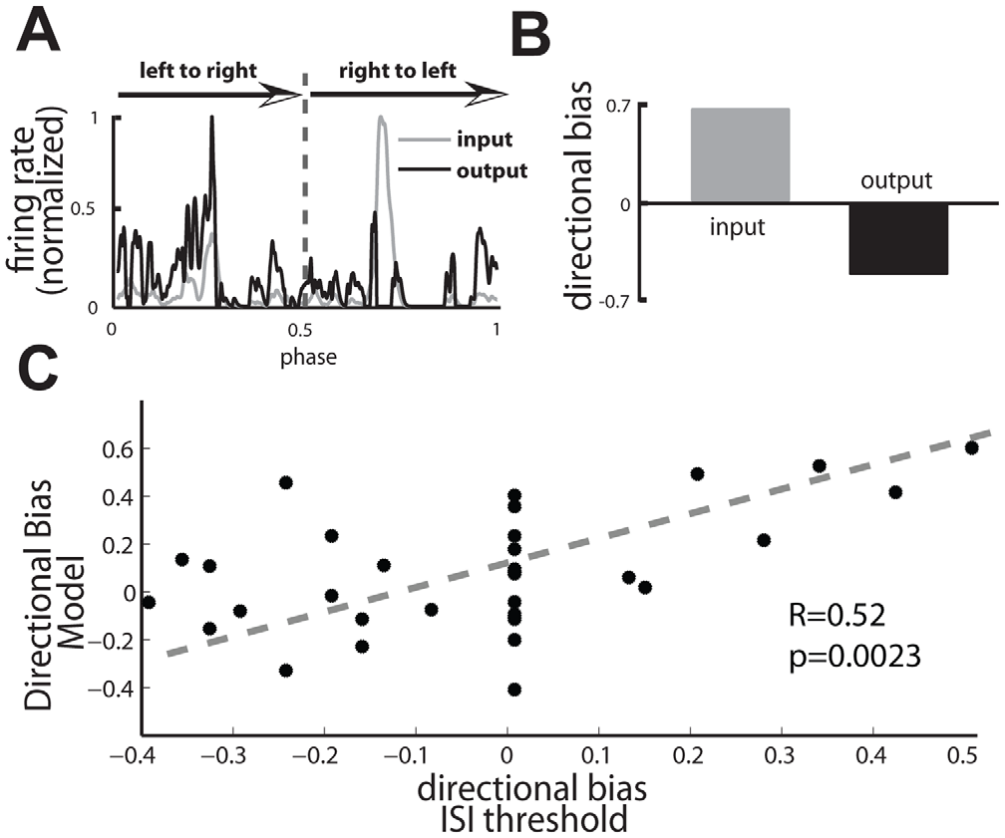
Comparison of a biologically plausible model with the ISI threshold. **A**) Input PSTH (gray) and output PSTH (black) from the model when the input consists of the full spike train from an example TS neuron. **B**) Output directional bias (black) and input directional bias (gray) computed from the PSTHs in C. Note the difference in sign. **C**) Directional bias of isolated spikes computed from the decoding model as a function of the directional bias of isolated spikes computed from the ISI threshold criterion. There was a significant positive correlation between both quantities (R = 0.52, p = 0.0023, N = 32). Parameter values used were τ_F_ = 70 msec, τ_D_ = 500 msec, τ_E_ = 5 msec, τ_I_ = 8 msec, G_I_ = 7, I_0_ = 3.41 msec, t_d_ = 4 msec.

We next tested whether the extracted isolated spikes could indeed code for the opposite movement direction than that coded by both the burst and full spike trains, as observed using an ISI threshold. As such, we used the spiketrain from the example neuron shown in Fig. 9B as an input to the model. We found that the input and output PSTHs were maximal for opposite movement directions (Fig. 12A) and thus displayed opposite directional biases (Fig. 12B). Finally, we computed the directional bias of isolated spikes obtained with our model against that computed from isolated spikes obtained with the ISI threshold criterion across our experimental dataset (Fig. 12C) and observed a significant positive correlation between both quantities (R = 0.52, p = 0.0023, N = 32). These results show that a generic circuit with a temporal delay can be used to selectively extract directional information carried by isolated spikes.

## Discussion

### Summary of results

We have explored the coding of movement direction by specific action potential patterns, namely bursts and isolated spikes, in a biophysical model of directional selectivity in midbrain neurons of weakly electric fish. We found that, for a wide range of parameter values, bursts displayed strong directional selectivity and isolated spikes displayed little or no directional selectivity consistent with previous findings [26]. However, we also found a qualitatively different regime for which bursts and isolated spikes were preferentially elicited when the object moved in opposite directions. As such, our results show for the first time that bursts and isolated spikes can code for opposite movement directions. We have also shown that subthreshold T-type calcium channels can greatly enhance the range of parameter values for which this regime was observed. This is because such channels must be de-inactivated by inhibition in order to be activated by subsequent excitation and give rise to a burst of action potentials. We have also shown experimental recordings from TS neurons in weakly electric fish for which bursts and isolated spikes coded for opposite movement directions. Finally, we have shown that plausible simple neural circuits can reliably extract isolated spikes from spike trains that consist of both bursts and isolated spikes. To our knowledge, these results constitute the first demonstration that bursts and isolated spikes can both code for movement direction in the same neuron. The relative simplicity and generality of our mathematical model suggests that our results will be applicable to other systems.

### Role of active burst dynamics in generating directional selectivity

Previous studies have shown that, for most TS neurons, bursts and isolated spikes were the most and least reliable indicators of motion direction, respectively [26]. Therefore, it was suggested that isolated spikes coded for stimulus attributes other than motion direction. In this study we have shown that, for some TS neurons, bursts and isolated spikes can code for opposite movement directions. Our model predicts that an active burst mechanism mediated by a T-type calcium conductance is not necessary in order to observe opposite coding of movement direction by bursts and isolated spikes. Nevertheless, this active burst mechanism greatly extended the range of parameter values for which we observed this regime and moreover increased the degree of directional selectivity as quantified by the directional bias associated with the burst and isolated spike trains to values that were observed experimentally. The fact that we only observed this regime in a few TS neurons suggests that such neurons are quite rare, which most likely explains why these neurons were not found in previous studies [26]. Further studies using intracellular recordings are needed in order to test whether these neurons constitute a specific class within the TS that would thus be distinct from neurons for which bursts and isolated spikes code for the same movement direction and whether they selectively express T-type calcium channels as predicted from our model.

### Functional relevance of opposite directional selectivity of bursts and isolated spikes

What is the functional relevance of having bursts and isolated spikes encode opposite movement direction in the same neuron? We propose that such parallel encoding may be used to discriminate different objects moving in opposite directions within the neuron's receptive field. Such parallel coding is entirely consistent with an emerging general picture in which bursts and isolated spikes can code for different stimulus attributes simultaneously and in parallel in the same neuron [25], [39], [41], [42], [44], [52], [66], [67]. In weakly electric fish, foreground and background motion in opposite directions could occur during prey capture [10] or during tracking behavior [5] and the simultaneous encoding of both fore and background movement may be necessary for proper motor control.

### Extracting bursts and isolated spikes

Our results are consistent with a growing body of literature that shows that bursts and isolated spikes can encode different stimulus attributes and thus might serve different functions [25], [41], [42], [44], [66], [68], [69]. This assumes that downstream neural circuits can somehow extract bursts and isolated spikes from a spike train. While previous studies have considered neural circuits that can selectively extract bursts [26], [41], [51], [70], we are not aware of any previous studies that have proposed biophysically plausible neural circuits that would be sensitive exclusively to isolated spikes prior to this one.

Specifically, we have proposed that the neural circuits that would respond exclusively to isolated spikes need to include a delay. This delay is necessary because any given spike cannot be unambiguously assigned as being part of a burst or being isolated without knowing at what time the next action potential will occur. Thus, it is necessary to compare the spike train at the present with the same spike train delayed by a time interval on the order of the burst threshold.

We note that neural circuits that use temporal combinations of delayed excitation and inhibition in order to achieve response selectivity have been described in other midbrain circuits and may be a general feature of sensory processing [63], [64], [71], [72]. In *Apteronotus leptorhynchus*, many TS neurons project to the optic tectum (OT) where neurons respond selectively to moving objects in a directionally biased fashion [4], [73]. It is possible that plasticity at the TS-OT synapses or a combination of excitation and inhibition from TS might enable OT neurons to decode bursts and/or isolated spikes from TS neurons. Future studies should investigate this interesting possibility.

### Implications for other systems

Our results show that the traditional method for measuring directional selectivity, in which the maximum firing rates elicited in response to the moving object in each direction are compared, can in some cases fail to capture salient information transmitted by direction selective neurons. This is because such techniques take the full spike train into account. Indeed, we found parameter regimes for which the isolated and full spike trains displayed selectivity for opposite movement directions. Moreover, for subsets of these parameters, the full spike train displayed little directional selectivity but for which the burst and isolated spike trains displayed opposite directional selectivity (see e.g. Fig. 6).

This result may have important consequences for the generation of direction selectivity in the mammalian visual cortex. Indeed, the electrosensory system has many parallels with thalamocortical pathways [74]. In particular, thalamic relay neurons within the lateral geniculate nucleus (LGN) have subthreshold T-type calcium channels that mediate burst firing [45], [59], [60], [75]–[80]. The spike trains from thalamic relay neurons consist of a mixture of bursts and isolated spikes in the awake-behaving animals [42], [81], [82]. While previous studies have shown that these neurons are not directionally selective [12], these did not consider action potential patterns such as bursts and isolated spikes. We hypothesize that bursts of action potentials from thalamic relay neurons in LGN carry specific directional information that is then used by postsynaptic neurons within the primary visual cortex to generate directionally biased responses. This hypothesis is supported by the fact that thalamocortical synapses display strong depression and that sustained isolated action potential firing from thalamic relay neurons activates this depression [45], [60], [83]. Nevertheless, ∼100 ms of inhibition can remove this depression as well as deinactivate T-type calcium channels. A subsequent depolarization caused by excitation can thus cause burst firing as well as an amplified post-synaptic response [45], [60], [83]. Studies performed within the LGN are necessary to validate this hypothesis and are beyond the scope of this paper.

### Conclusion

We investigated whether action potential patterns such as bursts and isolated spikes encoded movement direction in a model of directional selectivity in electrosensory midbrain neurons. We found parameter regimes in which bursts and isolated spikes could encode opposite movement directions in the same neuron even though the full spike train displays little or no directional selectivity. As such, neurons that are categorized as non-directionally selective using the full spike train may in fact be highly directional selective if one considers instead particular action potential patterns. Such coding of opposite movement directions by bursts and isolated spikes could be used in discriminating different objects moving in opposite directions within the neuron's receptive field and is likely to be found across sensory systems.

## Materials and Methods

### Ethics statement

McGill University's institutional Animal Care and Use Committee approved all experimental procedures and animal husbandry.

### Animals

We used the weakly electric fish *Apteronotus leptorhynchus* in this study. Animals were obtained from tropical fish suppliers and were housed in laboratory tanks for several days in order to become acclimated to the new environment. This was performed according to published guidelines [84]. The surgical and experimental procedures have been described in detail elsewhere [18], [35], [62], [85]–[88].

### Stimulation and recording

Extracellular recordings from TS neurons were made using previously described techniques [18], [35], [62], [89]. We used both patch [62], [89] and metal-filled micropipettes [62], [90]–[92] to obtain these recordings. The stimulus consisted of a 1.8 cm wide metal plate coated with a plastic coating on the side opposite to the animal that was actuated using a pen plotter (HP 7010B). This object moved back and forth along the animal's rostro-caudal axis over a distance of 20 cm [17], [18], [35], [93], [94] for at least 30 cycles. The sinusoid was centered at the animal's midpoint and had a frequency of 0.25 Hz, corresponding to an average velocity of ∼10 cm/sec. These velocities correspond to those that the animal experiences during prey capture [10] and within the velocities of error signals observed during refuge tracking [5].

Data were acquired with a Cambridge Electronic Design Power1401 hardware and Spike2 software (Cambridge, UK) and analyzed using Spike2 (CED) and custom-made routines in MATLAB (The Mathworks, Natick, MA). The recorded membrane potentials were thresholded in order to obtain the action potential times. We excluded neurons whose total spike count was less than 400 over the stimulus duration. Recorded spike trains were segregated into bursts and isolated spikes as described above using an ISI threshold. Neurons with burst or isolated spike counts less than 100 were not analyzed.

### Burst and isolated spike classification

We used an interspike interval threshold to separate the simulated spiking responses into burst and isolated spikes [25], [36], [41], [44] (Fig. 1D). Specifically, two consecutive action potentials that were separated by a time interval less than the burst threshold were considered as part of a burst. Spikes that were not part of bursts were included in the isolated spike train. The burst threshold was computed as the time at which the falling phase of initial peak of the autocorrelogram crossed the 99.9% Poisson confidence limit as done previously [25], [26], [36], [95].

### Quantifying directional selectivity and opposite directionality

The full spike, burst (i.e. the train of spikes that belong to bursts) and the isolated (i.e. the train of spikes that are isolated) spike trains were each used to compute peri-stimulus time histograms (PSTHs) in response to the moving object. We then computed a measure of directional bias as [18], [35]:
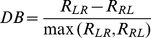
where *R_LR_*, *R_RL_* are the maximum firing rates obtained when the object moves from “left to right” and “right to left”, respectively (note that “left to right” corresponds to the object moving from the animal's snout to the tail and that “right to left” corresponds to the object moving from the tail to the snout) and max(*R_LR_, R_RL_*) is the maximum of the two. This measure varies between −1 and 1. DB values of 1 and −1 indicate complete direction preference for movement from left to right and from right to left, respectively, while a value of 0 indicates no direction selectivity.

To quantify the opposite directionality we used the directional biases computed from burst spikes and isolated spikes and then computed the opposite directionality index as:
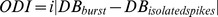
where i is 1 if the maximum firing rate of burst spikes and isolated spikes happen preferentially for the same object movement direction and is −1 otherwise. i is 0 if directional biases of bursts or isolated spikes equal 0.

### Modeling TS neurons

Our model TS neuron's one-dimensional receptive field consists of two 10 mm long adjacent ‘ON’ and ‘OFF’ zones. The ‘ON’ zone represents the output of E-type ELL pyramidal cells that are excited by the stimulus while the ‘OFF’ zone represents the output of I-type ELL pyramidal cells that are inhibited by the stimulus as observed experimentally [85], [96]. Then a point object moved at a speed of 10 cm/s back and forth across these zones. The output of each zone is then given by [18]:
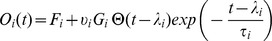
where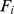 is the bias current which represents the baseline activity from E and I-type pyramidal cells which are approximately equal on average [97], [98] and ν_i_ = 1,−1 for i = ON, OFF, respectively. Here τ_i_ is the depression time constant associated with zone i, λ_i_ is the time that object enters zone i, and G_i_ is the gain of zone i. The responses of each zone were then convolved with an alpha function with time constant 20 msec to mimic synaptic EPSPs. Consistent with anatomical data showing that both E and I-type ELL pyramidal neurons make excitatory connections onto TS neurons [99], the input I(t) to our neuron model is taken to be:



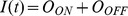



We note that the outputs from the ON and OFF zone, *O_ON_* and *O_OFF_*, were not delayed with respect to one another, which is consistent with recent experimental results showing no significant delay between the inputs from E and I-type sources onto TS neurons [88]. The TS neuron was modeled using the Hodgkin-Huxley formalism based on available experimental data [35], the model contains spiking sodium, delayed rectifier potassium, low threshold calcium (T-type), and leak conductances:
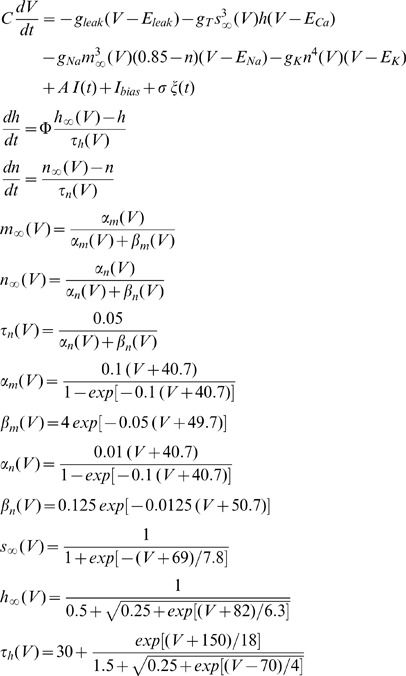
where C is the membrane capacitance, V is the transmembrane potential difference, g_leak_ is the leak conductance with reversal potential E_leak_. Here g_T_, g_Na_, and g_K_ are the voltage-gated calcium, sodium, and potassium conductances with reversal potentials E_Ca_, E_Na,_ and E_K_, respectively. A is the synaptic weight and I_bias_ is a constant bias current, σξ(t) is zero mean low-pass filtered Gaussian white noise with standard deviation σ that mimics sources of synaptic input [100].

We simulated this model numerically using an Euler-Maruyama Algorithm with integration time step dt = 0.0025 msec. Other parameter values used, unless otherwise stated, were g_leak_ = 0.18 μS, g_T_ = 0.32 μS, g_Na_ = 30 μS, g_K_ = 10 μS, E_leak_ = −65 mV, E_Ca_ = 120 mV, E_Na_ = 60 mV, E_K_ = −85 mV, C = 1 μF, A = 0.75, B = 0.1, = 2, G_1_ = G_2_ = 1, I_bias_ = −1.3 nA, G_ON_ = G_OFF_ = 1, F_ON_ = F_OFF_ = 2, τ_ON_ = 5 msec, τ_OFF_ = 500 msec. These values are comparable to those used in previous modeling studies [35], [59]. For some simulations, we set g_T_ = 0 and I_bias_ = 3.1 nA to adjust for firing rate. All simulations for computing PSTHs and directional biases were done over 1000 trials. We explored the parameter spaces by systematically varying synaptic depression time constants of ON and OFF zones in a range of 5 msec to 500 msec which is biologically relevant [18]. To explore the effect of synaptic depression time constants and bias current together we used synaptic depression time constants ratio τ_OFF_/τ_ON_ in the range of 1/50 to 50 in which τ_OFF_ and τ_ON_ were (in sec) [0.01 0.5], [0.01 0.4], [0.01 0.3], [0.01 0.3], [0.01 0.2], [0.01 0.1], [0.01 0.05], [0.01 0.04], [0.01 0.03], [0.01 0.02], [0.01 0.01], [0.02 0.01], [0.03 0.01], [0.04 0.01], [0.05 0.01], [0.1 0.01], [0.2 0.01], [0.3 0.01], [0.4 0.01], [0.5 0.01].

In all our analysis and figures in which the directional biases from our model were plotted as a function of parameters, directional biases whose magnitude was below 0.15 were set to zero. This is because previous analysis has shown that such directional biases were not significantly different from zero [18]. The burst threshold that was used for our model simulations was set at 10 msec as done previously [26].

### Modeling biophysically plausible mechanisms to extract isolated spikes

While the interspike interval threshold procedure described above is a simple computational method for segregating bursts and isolated spikes, it is not clear how such a threshold mechanism could be implemented in CNS circuits. A neural circuit which responses to bursts and is insensitive to isolated spikes has been previously considered [26]. However, the complement problem of designing a neural circuit that would be unresponsive to bursts but sensitive to isolated spikes has, to our knowledge, not been considered before.

Here we introduce a plausible circuit that can extract isolated spikes. Specifically, we consider the presynaptic spike train as a sum of delta functions:



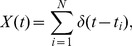
where t_i_ is the i^th^ spike time. X(t) is first passed through two parallel synapses. The first is excitatory and does not have any synaptic dynamics (i.e. no plasticity and the amplitude of the output EPSP is the same for all presynaptic action potentials), the output of this synapse is thus given by convolving the input spike train X(t) with an alpha function with time constant τ_E_:



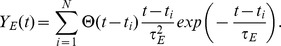



The second synapse is inhibitory and displays plasticity. This plasticity is described by facilitation and depression terms [101−104]:
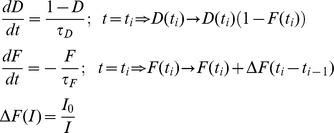



At the time of an input spike t_i_, D is first decreased by an amount F(t_i_)D(t_i_); then F is updated by an increment ΔF. The increment ΔF is inversely proportional to the time interval between the current action potential and the last one. As such, short time intervals such as those that occur during burst firing will cause more potentiation than longer ones. We have also introduced an upper bound for F (i.e. F(t)≤1) to prevent negative values for the update factor of the depression variable. The output of this synapse is thus given by:



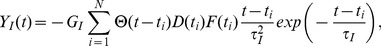
where D, F are the depression and facilitation terms, respectively. Here τ_I_ is the time constant of the alpha function that models the time course of the IPSP and G_I_ is a gain term. As such, the inhibitory synapse displayed strong facilitation in response to a burst of presynaptic action potentials. We assume that the output Y_E_(t) is delayed by a time t_d_. The postsynaptic output is then given by:



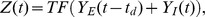



with TF defined as:
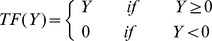



The post-synaptic spike train was obtained by thresholding Z(t) (i.e. finding the times at which Z(t) crosses a threshold value from below). We then took experimentally recorded spike sequences, and segregated them into bursts and isolated spikes using both our decoding model and ISI threshold methods. Then, we compared the sequences of burst and isolated spikes obtained from each model in the following way. We used signal detection theory [65] in order to quantify the decoding model's performance at detecting isolated spikes as defined by the ISI threshold. We computed the probability of correct detection (P_D_) as the fraction of spike times deemed to be part of isolated spike train according to the decoding model that were also deemed part of isolated spike train using the ISI threshold criterion (i.e. that were “correctly” classified). The probability of false alarm (P_FA_) was computed as the fraction of spike times deemed to be part of isolated spike train according to the decoding model that were deemed to be burst using the ISI threshold criterion (i.e. that were “incorrectly” classified). The overall performance can then be quantified by computing the probability of correct classification (PCC) as:
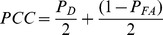



A value of PCC = 0.5 implies that our model performs at chance level compared to the ISI threshold criterion (i.e. that any given spike is randomly assigned as being part of a burst or isolated). In contrast, PCC = 1 indicates that the model performs identically to the ISI threshold criterion. We note that this does not imply that the ISI threshold criterion is optimal in any way as segregating bursts and isolated spikes, merely that our biophysically plausible decoding model performs as well. As such, signal detection theory is used here to determine how well the decoding model performs relative to the ISI threshold criterion.

## Supporting Information

Figure S1 **The gains G_ON_ and G_OFF_ strongly influence movement direction coding by bursts and isolated spikes.** **A**) Directional bias computed from the full spike train as a function of G_ON_ and G_OFF_. **B**) Directional bias computed from the burst spike train as a function of G_ON_ and G_OFF_. **C**) Directional bias computed from the isolated spike train as a function of G_ON_ and G_OFF_. **D**) Opposite direction selectivity index (ODI) as a function of τ_ON_ and τ_OFF_.
(TIF)

Figure S2 **The gains G_ON_ and G_OFF_ influence movement direction coding by bursts and isolated spikes with g_T_ = 0.** **A**) Directional bias computed from the full spike train as a function of G_ON_ and G_OFF_. **B**) Directional bias computed from the burst spike train as a function of G_ON_ and G_OFF_. **C**) Directional bias computed from the isolated spike train as a function of G_ON_ and G_OFF_. **D**) Opposite direction selectivity index as a function of G_ON_ and G_OFF_.
(TIF)
