# Supplementary figures and images for: Bursts and Isolated Spikes Code for Opposite Movement Directions in Midbrain Electrosensory Neurons
Source: PLoS One. 2012 Jun 29;7(6):e40339. doi: 10.1371/journal.pone.0040339 (PMC3386997; doi:10.1371/journal.pone.0040339)

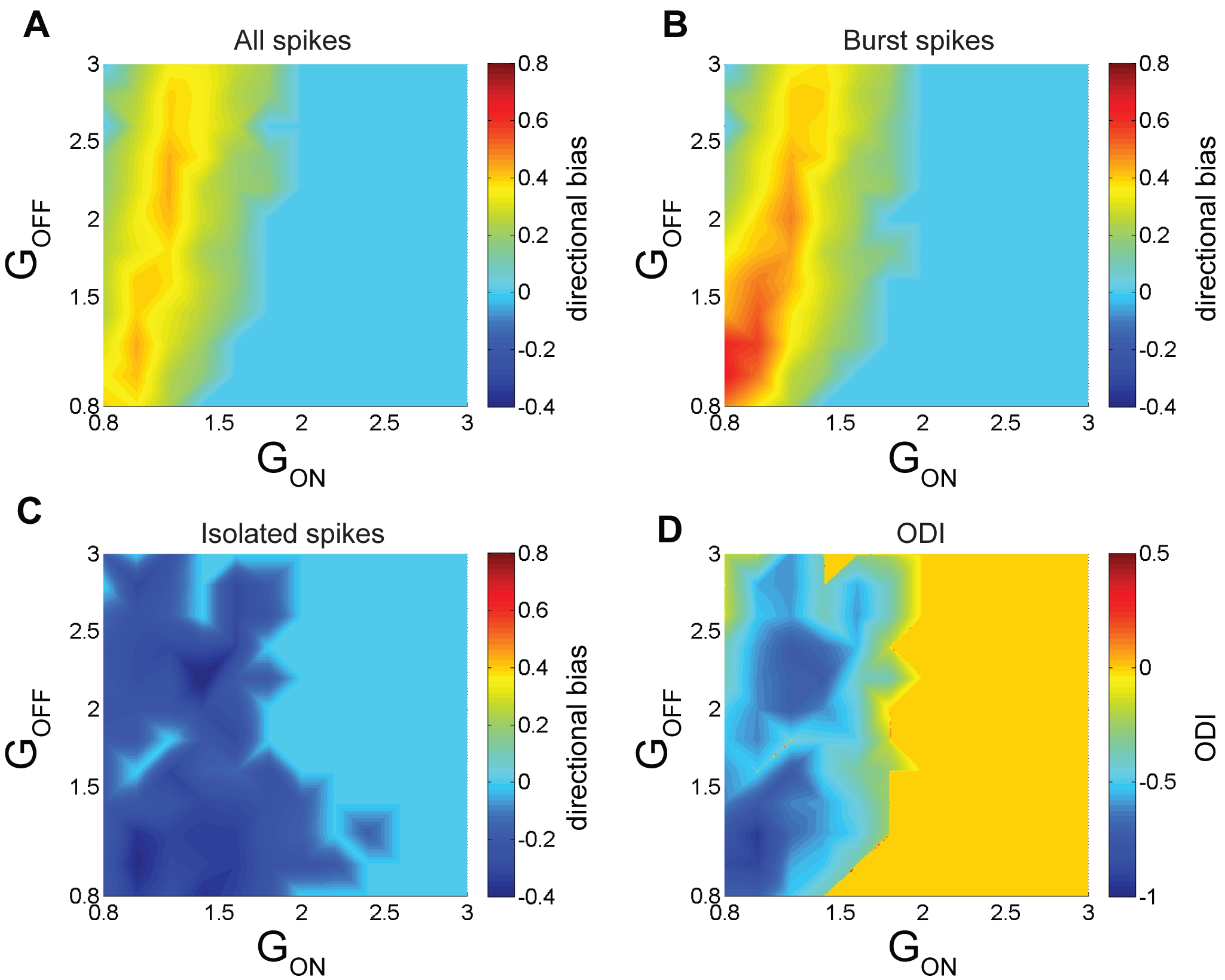

Supplement: Figure S1 — The gains GON and GOFF strongly influence movement direction coding by bursts and isolated spikes. A) Directional bias computed from the full spike train as a function of GON and GOFF. B) Directional bias computed from the burst spike train as a function of GON and GOFF. C) Directional bias computed from the isolated spike train as a function of GON and GOFF. D) Opposite direction selectivity index (ODI) as a function of τON and τOFF. (TIF) [file pone.0040339.s001.tif]

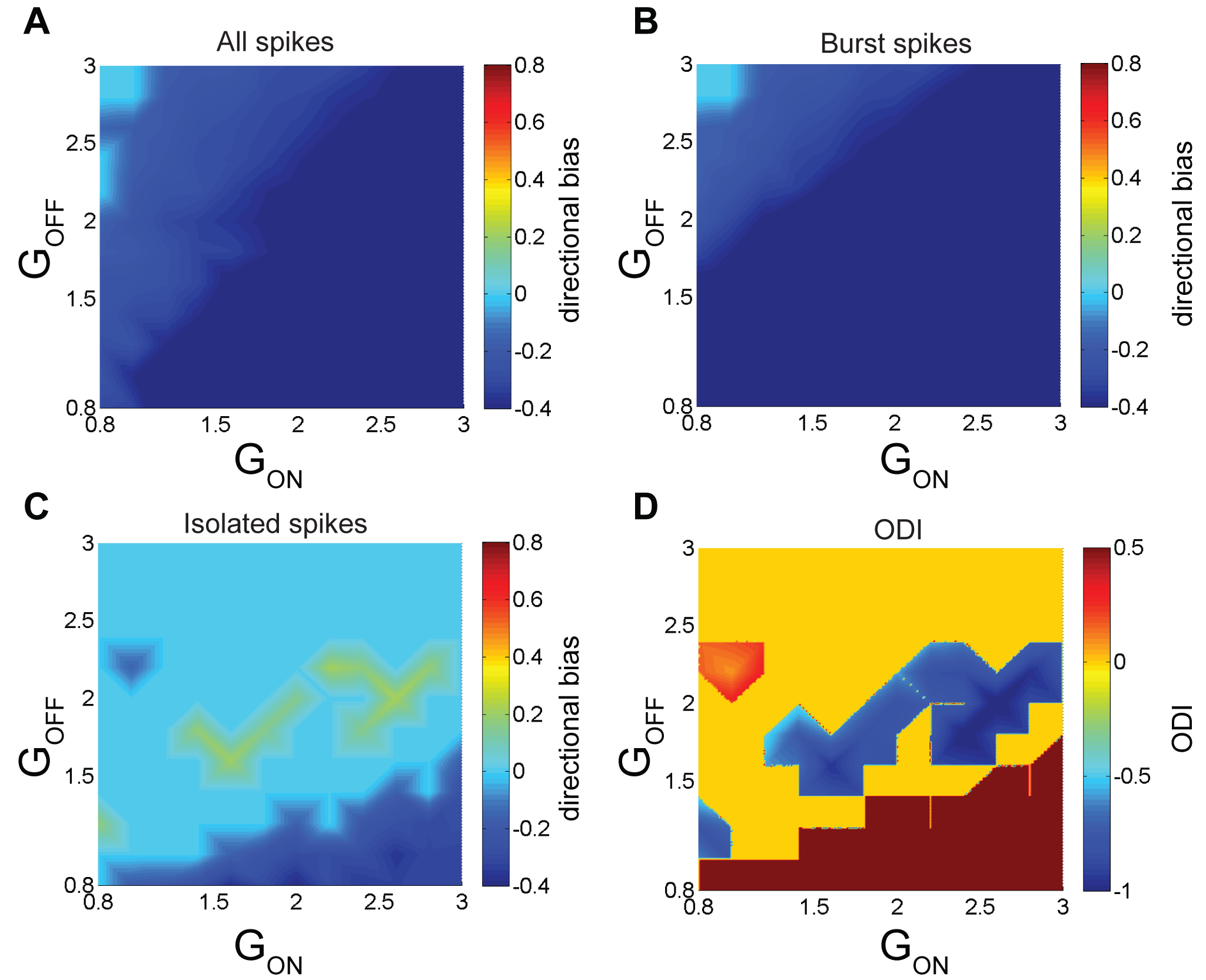

Supplement: Figure S2 — The gains GON and GOFF influence movement direction coding by bursts and isolated spikes with gT = 0. A) Directional bias computed from the full spike train as a function of GON and GOFF. B) Directional bias computed from the burst spike train as a function of GON and GOFF. C) Directional bias computed from the isolated spike train as a function of GON and GOFF. D) Opposite direction selectivity index as a function of GON and GOFF. (TIF) [file pone.0040339.s002.tif]
